# Developments in the classification and nomenclature of arthropod-infecting large DNA viruses that contain *pif* genes

**DOI:** 10.1007/s00705-023-05793-8

**Published:** 2023-06-14

**Authors:** Monique M. van Oers, Elisabeth A. Herniou, Johannes A. Jehle, Peter J. Krell, Adly M.M. Abd-Alla, Bergmann M. Ribeiro, David A. Theilmann, Zhihong Hu, Robert L. Harrison

**Affiliations:** 1 Laboratory of Virology, Wageningen University and Research, Wageningen, the Netherlands; 2 Institut de Recherche sur la Biologie de l’Insecte, UMR 7261, CNRS - University of Tours, 37200 Tours, France; 3 Institute for Biological Control, Julius Kühn-Institut, 69221 Dossenheim, Germany; 4 Department of Molecular and Cellular Biology, University of Guelph, Guelph, N1G 2W1 Canada; 5 Joint FAO/IAEA Programme of Nuclear Techniques in Food and Agriculture, Vienna International Centre, Vienna, Austria; 6 Laboratory of Baculovirus, Cell Biology Department, University of Brasília, Brasília, Brazil; 7 Summerland Research and Development Centre, Agriculture and Agri-Food Canada, 4200 Highway 97, Box 5000, Summerland, BC V0H1Z0 Canada; 8 State Key Laboratory of Virology, Wuhan Institute of Virology, Chinese Academy of Sciences, Wuhan, 430071 P. R. China; 9 Invasive Insect Biocontrol and Behavior Laboratory, USDA-ARS, 10300 Baltimore Avenue, Bldg 007 Barc‑West, Beltsville, MD 20705 USA

## Abstract

Viruses of four families of arthropod-specific, large dsDNA viruses (the nuclear arthropod large DNA viruses, or NALDVs) possess homologs of genes encoding conserved components involved in the baculovirus primary infection mechanism. The presence of such homologs encoding *per os* infectivity factors (*pif* genes), along with their absence from other viruses and the occurrence of other shared characteristics, suggests a common origin for the viruses of these families. Therefore, the class *Naldaviricetes* was recently established, accommodating these four families. In addition, within this class, the ICTV approved the creation of the order *Lefavirales* for three of these families, whose members carry homologs of the baculovirus genes that code for components of the viral RNA polymerase, which is responsible for late gene expression. We further established a system for the binomial naming of all virus species in the order *Lefavirales*, in accordance with a decision by the ICTV in 2019 to move towards a standardized nomenclature for all virus species. The binomial species names for members of the order *Lefavirales* consist of the name of the genus to which the species belongs (e.g., *Alphabaculovirus*), followed by a single epithet that refers to the host species from which the virus was originally isolated. The common names of viruses and the abbreviations thereof will not change, as the format of virus names lies outside the remit of the ICTV.

**Supplementary Information:**

The online version contains supplementary material available at 10.1007/s00705-023-05793-8.

## Introduction

Arthropod-infecting large DNA viruses belonging to four families – *Baculoviridae*, *Nudiviridae*, *Hytrosaviridae*, and *Nimaviridae* – share a set of common features that separate them from other arthropod-infecting large dsDNA viruses (Table 1). These viruses have collectively been referred to as nuclear arthropod large DNA viruses (NALDVs) [31–33] to distinguish them from what was previously referred to as the nucleo-cytoplasmic large DNA viruses (NCLDVs; now in the phylum *Nucleocytoviricota*) [9, 35]. In this paper, we explain recent developments in the classification of the NALDVs in the class *Naldaviricetes* and the recently established binomial nomenclature for viruses in the order *Lefavirales*, accommodating three of these virus families.

**Table 1:** Characteristics of arthropod-specific large dsDNA virus families and subfamilies

Feature	NALDV families				Other virus (sub)families			
	*Baculoviridae*	*Nudiviridae*	*Hytrosaviridae*	*Nimaviridae*	*Polydnaviriformidae*	*Ascoviridae*	*Entomopoxvirinae*	*Betairidovirinae*
*pif* genes	+	+	+	+	+^1^	-	-	-
Circular genome	+	+	+	+	±^2^	+	-	-
Enveloped, rod-shaped nucleocapsids	+	+	+	+	+	±^3^	-	-
Nuclear site of replication	+^4^	+	+	+	+	+^4^	-	±
Occlusion bodies	+	±	-	-	-	-	+	

^1^ Only polydnaviriforms of the genus *Bracoviriform* contain *pif* gene homologs.

^2^ Polydnaviriform particles contain circular DNA molecules originating from the genome of the parasitoid wasp host, while virus-derived

sequences remain integrated in the wasp genome.

^3^ Virions of this family are variably described as allantoid (sausage-shaped), reniform (kidney-shaped), or bacilliform (rod-shaped).

^4^ For ascovirids and betabaculoviruses, the host cell nuclear membrane ruptures prior to the completion of replication and virion assembly.

### The *pif* genes as a signature for the new class *Naldaviricetes*

NALDVs contain genes that encode proteins collectively known as *per os* infectivity factors (abbreviated as PIFs; Table 2). The *pif* genes were originally discovered in the genomes of baculoviruses, in which they are required exclusively for oral infectivity in host insects [7, 29]. Sequencing and analysis of nudivirid, hytrosavirid, and nimavirid genomes showed that homologs of four *pif* genes (*pif0/p74, pif1, pif2*, and *pif3)* were conserved in the genome sequences of all of these viruses [1, 30]. More recently, a fifth *pif* gene (*pif5/odve56*) was added to this list of conserved genes [15]. Furthermore, PIF proteins are also found in the viriform particles formed in the calyx of female braconid wasps, which are classified in the genus *Bracoviriform* in the recently renamed family *Polydnaviriformidae* [5, 6, 23]. These expressed bracoviriform PIF proteins originate from the endogenization of an ancient nudivirus [5, 12, 27].

**Table 2:** Core genes conserved among members of the class *Naldaviricetes*. A number of genes are found in all naldaviricete families; other genes are conserved in lefaviral families only.

Core genes and their function		*Naldaviricetes*			
		*Baculoviridae*	*Nudiviridae*	*Hytrosaviridae*	*Nimaviridae*
***Per os *** **infectivity factors (** ***pifs*** )*	*pif0 /p74*	+	+	+	+
	*pif1*	+	+	+	+
	*pif2*	+	+	+	+
	*pif3*	+	+	+	+
	*pif4*	+	+		+
	*pif5 /odv-e56*	+	+	+	+
	*pif6*	+	+		
	*pif8 /vp91*	+	+		
**Viral transcription complex**	*lef-4*	+	+	+	
	*lef-8*	+	+	+	
	*lef-9*	+	+	+	
	*p47*	+	+		
	*lef-5*	+	+	+	
	*vlf-1*	+	+		
**DNA replication**	*Dnapol*	+	+	+	+
	*Helicase*	+	+	+	
**Nucleocapsid proteins**	*38k*	+	+		
	*vp39*	+	+		
	*p6.9*	+	+		
**Sulfhydryl oxidase**	*p33*	+	+	+	+
**Unknown function**	*ac81*	+	+	+	

*pif7 is not included here, as it is only conserved in lepidopteran-infecting baculoviruses (alphabaculoviruses and betabaculoviruses).

**Fig. 1:**
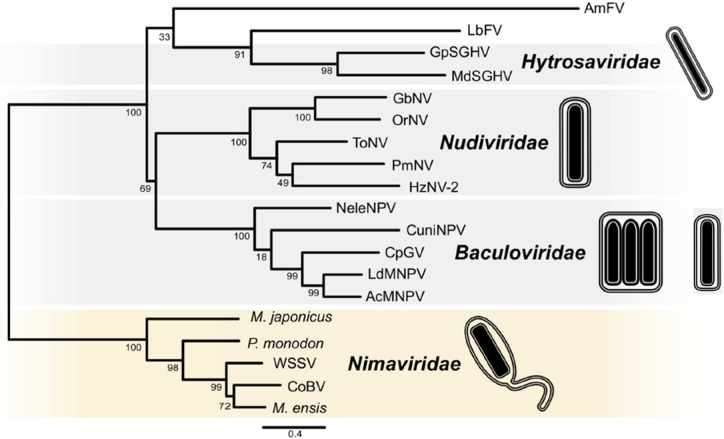
Phylogenetic analysis of members of the class *Naldaviricetes*. Concatenated alignments of five PIF amino acid sequences (*pif-0/p74*, *pif-1*, *pif-2*, *pif-3*, and *pif-5/odv-e56*), DNA polymerase (*dnapol*), and sulfhydryl oxidase (*p33*) were used to infer relationships by maximum likelihood as implemented in RAxML version 8.2.9 with substitution models and parameters selected for each alignment. Family-level classification is indicated for different clades in the midpoint-rooted tree. Abbreviations of virus names are as follows: AcMNPV, Autographa californica multiple nucleopolyhedrovirus; LdMNPV, Lymantria dispar MNPV; CpGV, Cydia pomonella granulovirus; CuniNPV, Culex nigripalpus nucleopolyhedrovirus; NeleNPV, Neodiprion lecontei NPV; OrNV, Oryctes rhinoceros nudivirus; GbNV, Gryllus bimaculatus NV; HzNV-2, Heliothis zea NV-2; PmNV, Penaeus monodon NV; ToNV, Tipula oleracea NV; GpSGHV, Glossina pallidipes salivary gland hypertrophy virus; MdSGHV, Musca domestica SGHV; AmFV, Apis mellifera filamentous virus; LbFV, Leptopilina boulardi FV; WSSV, white spot syndrome virus; CoBV, Chionoecetes opilio bacilliform virus. Endogenized nimaviruses from *Marsupenaeus japonicus*, *Peneaus monodon*, and *Metapeneaus ensis* were also included. This figure was reproduced and slightly modified from Kawato et al., 2019, J Virol 93:e01144-18, 10.1128/JVI.01144-18, with permission from the authors and the American Society for Microbiology.

Homologs of *pif* genes have not been identified in other viruses and thus are signature genes for members of the NALDV families. The conservation of *pif* genes in NALDVs, along with other shared genomic and phenotypic characteristics, such as their rod-shaped nucleocapsids and large double-stranded circular DNA genomes that replicate in the nucleus of infected cells (Table 1), indicate a common evolutionary origin for these viruses. Consequently, it was suggested to create a higher taxon in which to classify the four NALDV families [31–33] as well as unassigned viruses that may share these features. This idea was further supported by the fact that phylogenetic analysis indicated that the NALDVs formed a monophyletic group, separate from the nucleocytoviricots [35]. Bipartite network analysis of dsDNA virus genes and genomes showed that NALDV genomes and the encoded core genes formed a well-supported supermodule, separate from other observed modules [13]. However, several other analytical methods have not grouped the NALDV families together [2, 36, 37], suggesting that a significant degree of genetic divergence exists between members of different NALDV families. Members of the three families in the order *Herpesvirales* also exhibit a high degree of genetic divergence [21], but these families are classified in the same order on the basis of shared virion structural features that allude to their common evolutionary origin [10]. Unlike the capsids of herpesvirals, the structure of the rod-like NALDV particles varies from family to family, with observable differences in dimensions (length and width), features (presence or absence of a tail and/or terminal nucleocapsid cap), and protein composition. Furthermore, sequencing of Apis mellifera filamentous virus (AmFV; currently unclassified) revealed that its genome contained homologs of the same five *pif* genes found in viruses of the other NALDV families [11], suggesting that this unclassified virus is also an NALDV. In sharp contrast to the rod-shaped capsids of the other NALDVs, the capsid of AmFV is a very long (> 3 µm), flexuous filament that is coiled into an envelope [3]. This observation illustrates that, in addition to genetic divergence, *pif*-homolog-containing large dsDNA viruses can exhibit a considerable degree of structural divergence. Also, Leptopilina boulardi filamentous virus (LbFV; Fig. 1) is currently unclassified but encodes homologs of the *pif* genes [20].

Recently, the ICTV approved the use of taxa above the rank of “order” for virus classification [26]. We took advantage of the newly introduced hierarchy for virus classification and in 2020 proposed a class to harbour the four NALDV families (Fig. 1) (https://ictv.global/ictv/proposals/2020.006D.R.Naldaviricetes.zip). We felt that this higher rank would allow for classification of families of arthropod-infecting large dsDNA viruses that are distinguished by the inheritance of *pif* gene homologs but otherwise exhibit considerable genetic and structural variability and would therefore not be adequately classified within a single order. Based on the abbreviation NALDV, the approved class is named *Naldaviricetes*. The unclassified filamentous viruses (AmFV, LbFV) also appear to belong to this class but await assignment to species. Below, we describe why several other large dsDNA viruses that share some characteristics with the viruses now classified as *Naldaviricetes* members are not included in this new class.

### Relationships to other taxa

#### *Polydnaviriformidae*

As indicated above, members of the genus *Bracoviriform* of the family *Polydnaviriformidae*, which infect arthropods, evolved from an ancient nudivirus that integrated its genome into the genomic DNA of an ancient parasitoid wasp [5]. The integrated nudivirus sequences in the wasp genome have retained and express *pif* genes in female calyx cells [8, 34]. However, the family *Polydnaviriformidae* also contains the genus *Ichnoviriform*, whose members evolved from the genome of a different, unidentified virus that integrated into parasitoid wasps of a different family [4, 19, 28]. Ichnoviriforms do not contain *pif* homologs and thus do not meet the criteria for classification in the proposed order *Naldaviricetes*. A future revision of the family *Polydnaviriformidae* would be needed to enable movement of the bracoviriforms to a taxon within the class *Naldaviricetes*, together with the *Nudiviridae*.

#### *Entomopoxvirinae, Betairidovirinae* and *Ascoviridae*

Members of these three (sub)families of large dsDNA viruses infect arthropods but lack *pif* homologs and have other features that distinguish them from viruses in the class *Naldaviricetes* (Table 1) [37]. Entomopoxvirins and betairidovirins possess linear genomes, which are partially or wholly synthesized in the cytoplasm of infected cells. Ascovirids have circular genomes whose replication is initiated in the nucleus, but they clearly share a more recent origin with viruses in the subfamily *Betairidovirinae* [24]. In 2020, the ICTV ratified a taxonomic proposal to create the order *Pimascovirales* for the families *Ascoviridae*, *Iridoviridae*, and *Marseilleviridae* [17]. The proposal also created a realm, *Varidnaviria*, in which the families of the order *Pimascovirales* together with the other nucleocytoviricots are classified. The distinguishing characteristic of viruses classified in the realm *Varidnaviria* is the occurrence of a virus hallmark gene encoding a vertical double jelly-roll major capsid protein (VDJ-MCP). The members of the *Naldaviricetes*, on the other hand, do not contain homologs encoding a VDJ-MCP, but they do have other “connector” genes that might link the “baculo-like” supermodule with the nucleocytoviricot-polinton supermodule in dsDNA virus gene/genome networks [13]. This observation suggests that naldoviricetes may form an ancient branch of the *Varidnaviria* that has evolved to use different proteins for capsid assembly [13, 16]. However, we are presently not proposing to place the class *Naldaviricetes* into the *Varidnaviria* hierarchy.

### Establishment of the order *Lefavirales* within the class *Naldaviricetes*

**Fig. 2:**
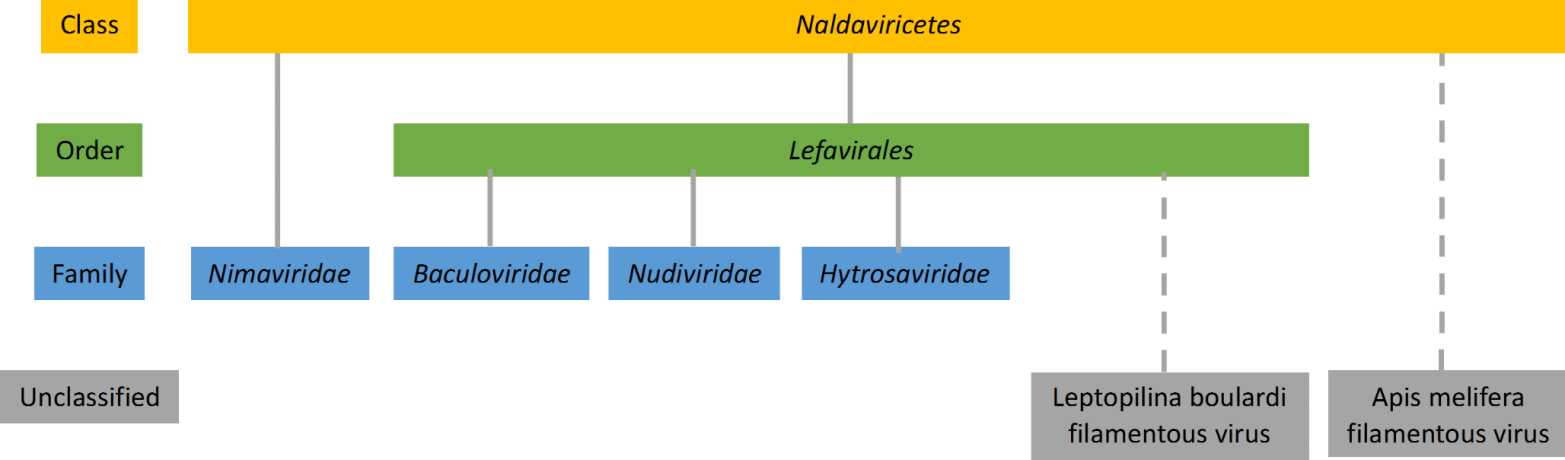
Taxonomic hierarchy of families of nuclear arthropod large DNA viruses. A new class, *Naldaviricetes*, was established for classification of the viruses in the four currently established families *Baculoviridae*, *Nudiviridae*, *Hytrosaviridae*, and *Nimaviridae. A* new order, *Lefavirales*, was introduced to include three of these families. The two viruses indicated at the top of the figure (Apis mellifera filamentous virus [3, 10] and Leptopilina boulardi filamentous virus [15]) are currently unclassified, but based on their genome content, they bear the hallmarks of members of the taxa *Naldaviricetes* and *Lefavirales*, respectively.

Phylogenies based on various naldaviricete sequence alignments often place viruses of the families *Baculoviridae*, *Nudiviridae*, and *Hytrosaviridae* into a clade separate from the *Nimaviridae* [6, 15, 29, 31]. Baculoviruses and viruses classified as members of the *Nudiviridae* and *Hytrosaviridae* contain homologs of genes that encode components of the baculovirus late-phase transcription complex, including three of the four subunits of the baculovirus DNA-directed RNA polymerase (*lef-4*, *lef-8*, and *lef-9*) (Table 2) [14, 22]. These homologs are not found in the genomes of nimavirids. We thus created an order within the class *Naldaviricetes* into which the families *Baculoviridae*, *Nudiviridae*, and *Hytrosaviridae* were placed (Fig. 2). This order is named *Lefavirales*, from the term “late expression factor” (abbreviated as *lef)*, which was previously coined to refer to genes identified in a screen for ORFs required for (or supporting) late-phase baculovirus transcription [22]. Lefavirals are characterized by the possession of conserved baculovirus transcription gene homologs and can also be distinguished from nimavirids in phylogenetic analysis (see Fig. 1). At present, we have refrained from creating an order for the family *Nimaviridae*, as there is insufficient information from which to extrapolate the distinguishing features of viruses in such an order. This strategy is consistent with the International Code of Virus Classification and Nomenclature (ICVCN; March 2021) Rule 3.2, which indicates that it is not mandatory to use all levels of the taxonomic hierarchy.

### Binomial naming system for virus species in the order *Lefavirales*

In 2021, the ICTV membership ratified a proposal (2018.001G.R) to adopt a binomial virus species naming system that follows the method of Linnaeus. This means that the Linnaean binomial format needs to be implemented for all virus species names, with a 2023 deadline. Accordingly, ICVCN Rule 3.21 now reads:

*"A species name shall consist of only two distinct word components separated by a space.*

*The first word component shall begin with a capital letter and be identical in spelling to the name of the genus to which the species belongs. The second word component shall not contain any suffixes specific for taxa of higher ranks. The entire species name (both word components) shall be italicized”.*

Since the order *Lefavirales* has three families, *Baculoviridae*, *Nudiviridae*, and *Hytrosaviridae*, it makes sense to name the species belonging to these families in a similar way. The two ICTV Study Groups concerned have therefore joined forces, designed a general method to convert all existing species names into binomial names, and submitted a formal ICTV taxonomic proposal in 2022 for consideration (https://ictv.global/ictv/proposals/2022.003D.Lefavirales_106rensp.zip). The same strategy is also used to assign species names to newly discovered lefavirals. Below, we present the new system and explain the reasoning behind the chosen method. As such, we aim to provide guidance for scientists in the field for naming new lefaviral species. In the following explanations, we will use baculovirids as examples. All updated lefaviral species names are provided in Table 3. Please be aware that the renaming only applies to virus species names. The names of viruses and their isolates remain unchanged. Since only names of virus species and higher taxa are regulated by the ICTV, it is expected that the way the viruses themselves are routinely named in the literature will remain unchanged. Thus, for naming old and new naldaviricete virus isolates, the historic conventions and practices should be continued as indicated in Table 3.

**Table 3:** List of currently valid lefaviral binomial species names along with common virus names and their abbreviations. Please note that virus names will remain unaffected by recent nomenclatural changes.

Family/genus	Binominal species name	Exemplar/common virus name	Abbreviation
**Family** ***Baculoviridae***			
*Alphabaculovirus*	*Alphabaculovirus adhonmai*	Adoxophyes honmai nucleopolyhedrovirus	AdhoNPV
	*Alphabaculovirus agipsilonis*	Agrotis ipsilon multiple nucleopolyhedrovirus	AgipNPV
	*Alphabaculovirus agsegetum*	Agrotis segetum nucleopolyhedrovirus A	AgseNPV-A
	*Alphabaculovirus alteragsegetum*	Agrotis segetum nucleopolyhedrovirus B	AgseNPV-B
	*Alphabaculovirus anpernyi*	Antheraea pernyi nucleopolyhedrovirus	AnpeNPV
	*Alphabaculovirus angemmatalis*	Anticarsia gemmatalis multiple nucleopolyhedrovirus	AgMNPV
	*Alphabaculovirus ardigrammae*	Artaxa digramma nucleopolyhedrovirus	ArdiNPV
	*Alphabaculovirus aucalifornicae*	Autographa californica multiple nucleopolyhedrovirus	AcMNPV
	*Alphabaculovirus bomori*	Bombyx mori nucleopolyhedrovirus	BmNPV
	*Alphabaculovirus busuppressariae*	Buzura suppressaria nucleopolyhedrovirus	BuzuNPV
	*Alphabaculovirus capomonae*	Catopsilia pomona nucleopolyhedrovirus	CapoNPV
	*Alphabaculovirus alterchofumiferanae*	Choristoneura fumiferana DEF multiple nucleopolyhedrovirus	CfMNPV-DEF
	*Alphabaculovirus chofumiferanae*	Choristoneura fumiferana multiple nucleopolyhedrovirus	CfMNPV
	*Alphabaculovirus chomurinanae*	Choristoneura murinana nucleopolyhedrovirus	ChmuNPV
	*Alphabaculovirus chorosaceanae*	Choristoneura rosaceana nucleopolyhedrovirus	ChroNPV
	*Alphabaculovirus chrychalcites*	Chrysodeixis chalcites nucleopolyhedrovirus	ChchNPV
	*Alphabaculovirus chrincludentis*	Chrysodeixis includens nucleopolyhedrovirus	ChinNPV
	*Alphabaculovirus clabilineatae*	Clanis bilineata nucleopolyhedrovirus	ClbiNPV
	*Alphabaculovirus covestigialis*	Condylorrhiza vestigialis nucleopolyhedrovirus	CovoNPV
	*Alphabaculovirus crypeltasticae*	Cryptophlebia peltastica nucleopolyhedrovirus	CrpeNPV
	*Alphabaculovirus cycundantis*	Cyclophragma undans nucleopolyhedrovirus	CyunNPV
	*Alphabaculovirus dijunonis*	Dione juno nucleopolyhedrovirus	DijuNPV
	*Alphabaculovirus ecobliquae*	Ectropis obliqua nucleopolyhedrovirus	EcobNPV
	*Alphabaculovirus eppostvittanae*	Epiphyas postvittana nucleopolyhedrovirus	EppoNPV
	*Alphabaculovirus eupseudoconspersae*	Euproctis pseudoconspersa nucleopolyhedrovirus	EupsNPV
	*Alphabaculovirus helarmigerae*	Helicoverpa armigera nucleopolyhedrovirus	HearNPV
	*Alphabaculovirus heleucae*	Hemileuca species nucleopolyhedrovirus	HespNPV
	*Alphabaculovirus hycuneae*	Hyphantria cunea nucleopolyhedrovirus	HycuNPV
	*Alphabaculovirus hytalacae*	Hyposidra talaca nucleopolyhedrovirus	HytaNPV
	*Alphabaculovirus lafiscellariae*	Lambdina fiscellaria nucleopolyhedrovirus	LafiNPV
	*Alphabaculovirus leseparatae*	Leucania separata nucleopolyhedrovirus	LeseNPV
	*Alphabaculovirus lonobliquae*	Lonomia obliqua nucleopolyhedrovirus	LoobNPV
	*Alphabaculovirus lydisparis*	Lymantria dispar multiple nucleopolyhedrovirus	LdMNPV
	*Alphabaculovirus lyxylinae*	Lymantria xylina nucleopolyhedrovirus	LyxyNPV
	*Alphabaculovirus mabrassicae*	Mamestra brassicae multiple nucleopolyhedrovirus	MbMNPV
	*Alphabaculovirus maconfiguratae*	Mamestra configurata nucleopolyhedrovirus A	MacoNPV-A
	*Alphabaculovirus altermaconfiguratae*	Mamestra configurata nucleopolyhedrovirus B	MacoNPV-B
	*Alphabaculovirus mavitratae*	Maruca vitrata nucleopolyhedrovirus	MaviNPV
	*Alphabaculovirus myunipunctae*	Mythimna unipuncta nucleopolyhedrovirus A	MyunNPV-A
	*Alphabaculovirus altermyunipunctae*	Mythimna unipuncta nucleopolyhedrovirus B	MyunNPV-B
	*Alphabaculovirus opbrumatae*	Operophtera brumata nucleopolyhedrovirus	OpbrNPV
	*Alphabaculovirus orleucostigmae*	Orgyia leucostigma nucleopolyhedrovirus	OrleNPV
	*Alphabaculovirus orpseudotsugatae*	Orgyia pseudotsugata multiple nucleopolyhedrovirus	OPMNPV
	*Alphabaculovirus oxochraceae*	Oxyplax ochracea nucleopolyhedrovirus	OxocNPV
	*Alphabaculovirus pesauciae*	Peridroma saucia nucleopolyhedrovirus	PesaNPV
	*Alphabaculovirus peluscae*	Perigonia lusca nucleopolyhedrovirus	PeluNPV
	*Alphabaculovirus ranus*	Rachiplusia nu nucleopolyhedrovirus	RanuNPV
	*Alphabaculovirus speridanae*	Spodoptera eridania nucleopolyhedrovirus 251	SperNPV-251
	*Alphabaculovirus altersperidanae*	Spodoptera eridania nucleopolyhedrovirus -CNPSo-165	SperNPV-CNPSo-165
	*Alphabaculovirus spexemptae*	Spodoptera exempta nucleopolyhedrovirus	SpexNPV
	*Alphabaculovirus spexiguae*	Spodoptera exigua multiple nucleopolyhedrovirus A	SeMNPV-A
	*Alphabaculovirus alterspexiguae*	Spodoptera exigua multiple nucleopolyhedrovirus B	SeMNPV-B
	*Alphabaculovirus spofrugiperdae*	Spodoptera frugiperda multiple nucleopolyhedrovirus	SfMNPV
	*Alphabaculovirus splittoralis*	Spodoptera littoralis nucleopolyhedrovirus	SpliNPV
	*Alphabaculovirus spliturae*	Spodoptera litura nucleopolyhedrovirus	SpltNPV
	*Alphabaculovirus sujujubae*	Sucra jujuba nucleopolyhedrovirus	SujuNPV
	*Alphabaculovirus thorichlaceae*	Thysanoplusia orichalcea nucleopolyhedrovirus	ThohNPV
	*Alphabaculovirus trini*	Trichoplusia ni single nucleopolyhedrovirus	TnSNPV
	*Alphabaculovirus urprotei*	Urbanus proteus nucleopolyhedrovirus	UrprNPV
	*Alphabaculovirus wisignatae*	Wiseana signata nucleopolyhedrovirus	WisiNPV
*Betabaculovirus*	*Betabaculovirus adoranae*	Adoxophyes orana granulovirus	AdorGV
	*Betabaculovirus agsegetum*	Agrotis segetum granulovirus	AgseGV
	*Betabaculovirus arrapae*	Artogeia rapae granulovirus	ArraGV
	*Betabaculovirus chofumiferanae*	Choristoneura fumiferana granulovirus	CfGV
	*Betabaculovirus clanachoretae*	Clostera anachoreta granulovirus	ClanGV
	*Betabaculovirus clanastomosis*	Clostera anastomosis granulovirus A	ClasGV-A
	*Betabaculovirus alterclanastomosis*	Clostera anastomosis granulovirus B	ClasGV-B
	*Betabaculovirus cnamedinalis*	Cnaphalocrocis medinalis granulovirus	CnmeGV
	*Betabaculovirus cryleucotretae*	Cryptophlebia leucotreta granulovirus	CrleGV
	*Betabaculovirus cypomonellae*	Cydia pomonella granulovirus	CpGV
	*Betabaculovirus disaccharalis*	Diatraea saccharalis granulovirus	DisaGV
	*Betabaculovirus epaporemae*	Epinotia aporema granulovirus	EpapGV
	*Betabaculovirus erellonis*	Erinnyis ello granulovirus	ErelGV
	*Betabaculovirus habrilliantis*	Harrisina brillians granulovirus	HabrGV
	*Betabaculovirus helarmigerae*	Helicoverpa armigera granulovirus	HearGV
	*Betabaculovirus hycuneae*	Hyphantria cunea granulovirus	HycuNPV
	*Betabaculovirus lacoleraceae*	Lacanobia oleracea granulovirus	LaolGV
	*Betabaculovirus molatipedis*	Mocis latipes granulovirus	MolaGV
	*Betabaculovirus myunipunctae*	Mythimna unipuncta granulovirus A	MyunGV-A
	*Betabaculovirus altermyunipunctae*	Mythimna unipuncta granulovirus B	MyunGV-B
	*Betabaculovirus maphaseoli*	Matsumuraeses phaseoli granulovirus	MaphGV
	*Betabaculovirus phoperculellae*	Phthorimaea operculella granulovirus	PhopGV
	*Betabaculovirus plinterpunctellae*	Plodia interpunctella granulovirus	PlinGV
	*Betabaculovirus pluxylostellae*	Plutella xylostella granulovirus	PlxyGV
	*Betabaculovirus spofrugiperdae*	Spodoptera frugiperda granulovirus	SfGV
	*Betabaculovirus spliturae*	Spodoptera litura granulovirus	SpltGV
	*Betabaculovirus trini*	Trichoplusia ni granulovirus	TnGV
	*Betabaculovirus xecnigri*	Xestia c-nigrum granulovirus	XecnGV
*Gammabaculovirus*	*Gammabaculovirus nelecontei*	Neodiprion lecontei nucleopolyhedrovirus	NeleNPV
	*Gammabaculovirus nesertiferis*	Neodiprion sertifer nucleopolyhedrovirus	NeseNPV
*Deltabaculovirus*	*Deltabaculovirus cunigripalpi*	Culex nigripalpus nucleopolyhedrovirus	CuniNPV
**Family** ***Nudiviridae***			
*Alphanudivirus*	*Alphanudivirus droinnubilae*	Drosophila innubila nudivirus	DiNV
	*Alphanudivirus dromelanogasteris*	Kallithea virus	KV
	*Alphanudivirus alterdromelanogasteris*	Tomelloso virus	TNV
	*Alphanudivirus tertidromelanogasteris*	Esparto virus	ENV
	*Alphanudivirus quartudromelanogasteris*	Mauternbach virus	MNV
	*Alphanudivirus grybimaculati*	Gryllus bimaculatus nudivirus	GbNV
	*Alphanudivirus oryrhinocerotis*	Oryctes rhinoceros nudivirus	OrNV
*Betanudivirus*	*Betanudivirus hezeae*	Heliothis zea nudivirus	HzNV
*Gammanudivirus*	*Gammanudivirus hogammari*	Homarus gammarus nudivirus	HgNV
	*Gammanudivirus pemonodonis*	Penaeus monodon nudivirus	PmNV
	*Gammanudivirus cracrangonis*	Crangon crangon nudivirus	CcNV
	*Gammanudivirus cameanadis*	Carcinas meneas nudivirus	CmPV
*Deltanudivirus*	*Deltanudivirus tipoleraceae*	Tipula oleracea nudivirus	ToGV
**Family** ***Hytrosaviridae***			
*Glossinavirus*	*Glossinavirus glopallidipedis*	Glossina pallipides salivery gland hypertrophy virus	GpSGHV
*Muscavirus*	*Muscavirus musdomesticae*	Musca domestica salivery gland hypertrophy virus	MdSGHV

### Issues with the previous lefaviral species naming system

In the past, the species names for lefavirals varied in format from family to family. For the family *Baculoviridae*, species names previously consisted of the binomial name of the host species, sometimes followed by a virion structural characteristic (“multiple” in *Autographa californica multiple nucleopolyhedrovirus*) and/or by a now obsolete genus name (*nucleopolyhedrovirus, granulovirus*). In most cases the virus species name did not differ from the virus name, except that the virus species name was fully written in italic letters, which made it often complicated to distinguish between the virus species and the virus itself. Nudivirid species names also started with the host species name, followed by the common name for viruses of this family (*nudivirus*). Species names in the family *Hytrosaviridae* consisted of the genus name of the host (e.g., *Glossina*) followed by the virus genus name (*hytrosavirus*). Species names for all three families featured part or all of the species names of the viral hosts. In developing specific epithets for lefaviral species names, we have retained this familiar feature in order to ease the transition to a new binomial format that is consistent among all lefaviral families.

The remaining family in the class *Naldaviricetes*, the *Nimaviridae*, was not included in this proposed binomial naming system, as the naming of the only classified species in this family is historically based on symptoms and not on host species, in contrast to lefaviral species, but it would be logical to follow the same principle for nimavirid species.

### Binomial naming method for virus species in the order *Lefavirales*

The binomial species names for lefavirals are composed as follows:

- As for Linnaean binomial species names in general, the first word of the species name is the name of the genus to which the virus species belongs, starting with a capital (e.g., *Alphabaculovirus* or *Betanudivirus*).
- The second word (the epithet) reflects the Latin species name of the arthropod host from which the virus was originally isolated. It is composed of the first 2–4 letters of the host genus directly coupled to the genitive form of the epithet of the host species name. For example, *Autographa californica multiple nucleopolyhedrovirus* is now *Alphabaculovirus aucalifornicae*, and *Cydia pomonella granulovirus* is now *Betabaculovirus cypomonellae.* The specific epithet should be readable and pronounceable.
- Latin ordinal prefixes are added to the specific epithet to distinguish between species with isolates originating from the same host. When a second species from the same host is identified, the prefix “*alter-*“ is placed at the start of the epithet. For example, *Mamestra configurata nucleopolyhedrovirus A*, the first species identified from the bertha armyworm, *Mamestra configurata*, is now *Alphabaculovirus maconfiguratae*, while *Mamestra configurata nucleopolyhedrovirus B*, the second species with isolates identified from *M. configurata*, became *Alphabaculovirus altermaconfiguratae*. For subsequent species to classify viruses isolated from the same host, the appropriate Latin ordinal prefixes will be added to the specific epithet. For example, “*tert(i)*” and “*quart(u)*” will be placed in front of “-*maconfiguratae*” if alphabaculoviruses representing a third and fourth distinct species from *M. configurata* would be identified.

### Explanations and examples of the epithet strategy

It might seem simpler to adapt the host-specific epithet alone as the specific epithet for lefaviral species, as was done for the microsporidium *Nosema ceranae*, a pathogen of the Asiatic honey bee, *Apis cerana*. However, this approach does not account for situations in which two distinct viruses from the same genus are isolated from different hosts that share the same specific epithet. There is already an example of this situation: in addition to Autographa californica multiple nucleopolyhedrovirus (already classified as *Alphabaculovirus aucalifornicae*), there is another alphabaculovirus identified from the California oakworm, *Phryganidia californica* [18]. Adding the 2–4 first letters of the host’s genus name as the start of the epithet resolves this problem.

The genus name, per definition, ends in “*-virus*”, and as a consequence, the genus names are all of neuter gender in Latin. We can therefore not simply use the original epithet of the host, which may have been of female, male, or neuter gender. Therefore, we proposed to use the genitive form of the epithet of the insect species in the binomial name of the virus species. Genitive forms have the meaning: “owned by, derived from, belonging to”. (The singular genitive form of most Latin words ends in -*ae*, *-i*, or *-is*, depending on the declension. All plural genitive forms end in -*um*). For explanations of less obvious epithets, see Supplementary Table S1.

In the situation where the host epithet already appears to be neuter, there would not be a strict linguistic need to change it. However, for overall consistency, we decided to use the genitive form there as well. Example: *Betabaculovirus xecnigri* (from the host *Xestia c-nigrum*). But what to do if the host’s epithet is already in the genitive form or looks the same as a genitive form? Then we will leave it as it is, as the use of double genitives is not useful. This is exemplified by *Alphabaculovirus anpernyi* (from the host *Antheraea pernyi*), *Betabaculovirus cnamedinalis* (from *Cnaphalocrocis medinalis*), *Betabaculovirus agsegetum* (already genitive plural in *Agrotis segetum;* from *“*seges”, meaning from the grain fields/crops).

In the past, a capital letter was appended to the end of baculovirus species names to distinguish species with isolates originating from the same host, as described above for alphabaculoviruses from *M. configurata.* One simple approach to reproducing this solution in the context of a binomial system might have been to attach the letter to the specific epithet of a binomial name with a hyphen. However, the use of hyphens to attach numbers or letters to the end of a series of species names is specifically excluded by ICVCN Rule 3.13. Thus, ordinal prefixes are used to distinguish different species isolated from the same host, as described above. The exact form of the ordinal prefixes will depend on the ease of pronunciation of the resulting epithet.

The background for the adopted strategy and some more general rules for composing epithets can be found in: “Advice and guidelines to Study Groups on the implementation of binomial species names", to be found at https://ictv.global/filebrowser/download/435 or in the recently published paper by Postler and collaborators [25].

## Conclusions

In this paper, we report recent major changes in the taxonomy of NALVDs, which are now part of the official ICTV taxonomy. The class *Naldaviricetes* and the order *Lefavirales* were established in 2021. The binomial species naming system for lefavirals was ratified in April 2023. In case of questions on how to name new virus species, please contact the respective ICTV Study Group. It is further proposed to continue using conventional naming and abbreviations for virus isolates, which will facilitate distinguishing between viruses and virus species.

### Electronic Supplementary Material

Below is the link to the electronic supplementary material

Supplementary Material 1
