# Correction to: Developments in the classification and nomenclature of arthropod-infecting large DNA viruses that contain *pif* genes

**DOI:** 10.1007/s00705-023-05857-9

**Published:** 2023-09-20

**Authors:** Monique M. van Oers, Elisabeth A. Herniou, Johannes A. Jehle, Peter J. Krell, Adly M. M. Abd-Alla, Bergmann M. Ribeiro, David A. Theilmann, Zhihong Hu, Robert L. Harrison

**Affiliations:** 1 Laboratory of Virology, Wageningen University and Research, Wageningen, The Netherlands; 2 Institut de Recherche sur la Biologie de l’Insecte, UMR 7261, CNRS-University of Tours, 37200 Tours, France; 3 Institute for Biological Control, Julius Kühn-Institut, 69221 Dossenheim, Germany; 4 Department of Molecular and Cellular Biology, University of Guelph, Guelph, N1G 2W1 Canada; 5 Joint FAO/IAEA Programme of Nuclear Techniques in Food and Agriculture, Vienna International Centre, Vienna, Austria; 6 Laboratory of Baculovirus, Cell Biology Department, University of Brasília, Brasília, Brazil; 7 Summerland Research and Development Centre, Agriculture and Agri-Food Canada, 4200 Highway 97, Box 5000, Summerland, BC V0H1Z0 Canada; 8 State Key Laboratory of Virology, Wuhan Institute of Virology, Chinese Academy of Sciences, Wuhan, 430071 People’s Republic of China; 9 Invasive Insect Biocontrol and Behavior Laboratory, USDA-ARS, 10300 Baltimore Avenue, Bldg 007 Barc-West, Beltsville, MD 20705 USA

**Correction to: Archives of Virology (2023) 168:182** 10.1007/s00705-023-05793-8

Legends for Fig. 1 and Fig. 2 were incorrectly published in the original version, in which these had been interchanged. This is corrected now (Figs. 1, 2).

**Fig. 1:**
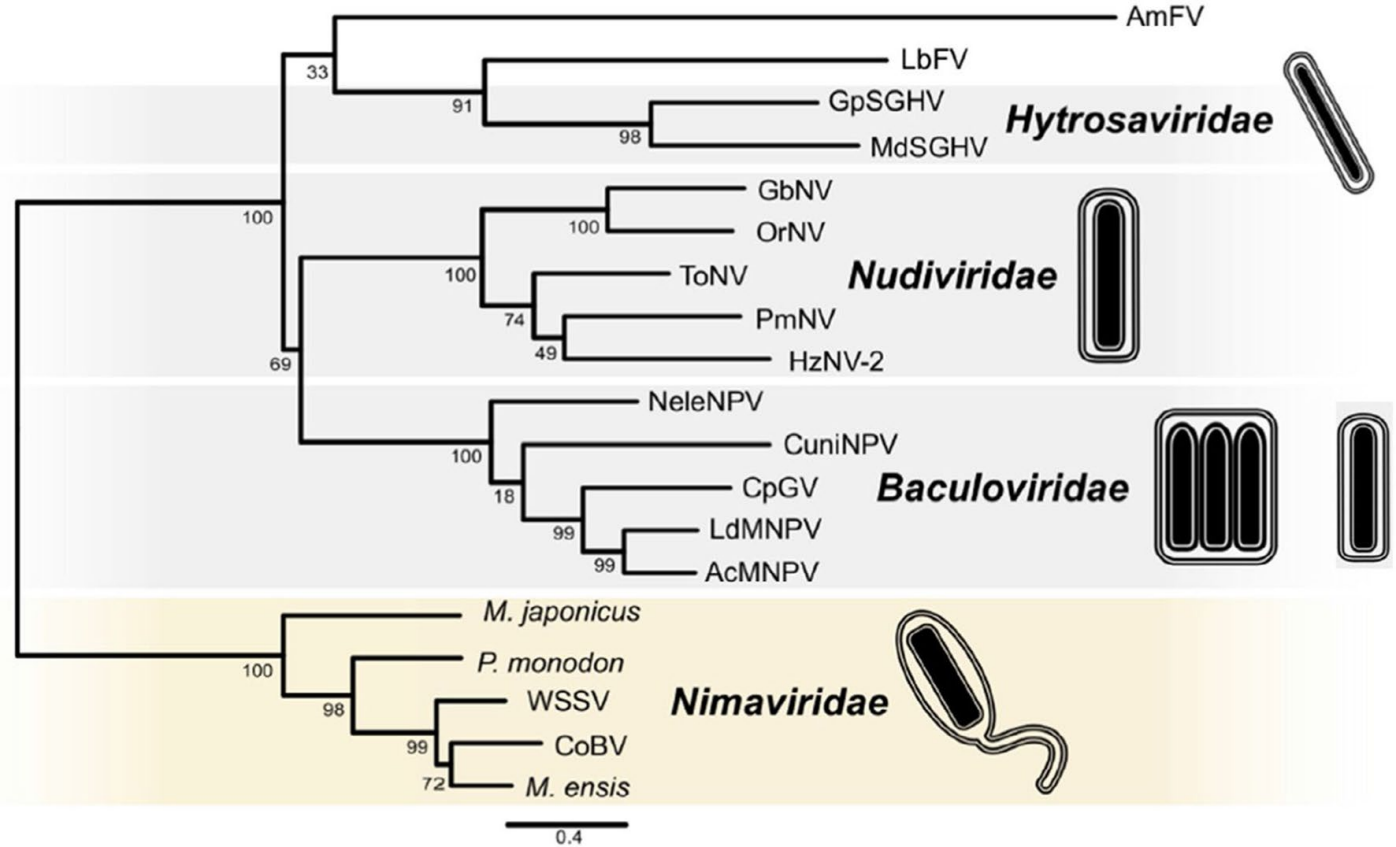
Phylogenetic analysis of members of the class *Naldaviricetes*. Concatenated alignments of five PIF amino acid sequences (*pif-0/p74*, *pif-1*, *pif-2*, *pif-3*, and *pif-5/odv-e56*), DNA polymerase (*dnapol*), and sulfhydryl oxidase (*p33*) were used to infer relationships by maximum likelihood as implemented in RAxML version 8.2.9 with substitution models and parameters selected for each alignment. Family-level classification is indicated for different clades in the midpoint-rooted tree. Abbreviations of virus names are as follows: AcMNPV, Autographa californica multiple nucleopolyhedrovirus; LdMNPV, Lymantria dispar MNPV; CpGV, Cydia pomonella granulovirus; CuniNPV, Culex nigripalpus nucleopolyhedrovirus; NeleNPV, Neodiprion lecontei NPV; OrNV, Oryctes rhinoceros nudivirus; GbNV, Gryllus bimaculatus NV; HzNV-2, Heliothis zea NV-2; PmNV, Penaeus monodon NV; ToNV, Tipula oleracea NV; GpSGHV, Glossina pallidipes salivary gland hypertrophy virus; MdSGHV, Musca domestica SGHV; AmFV, Apis mellifera filamentous virus; LbFV, Leptopilina boulardi FV; WSSV, white spot syndrome virus; CoBV, Chionoecetes opilio bacilliform virus. Endogenized nimaviruses from *Marsupenaeus japonicus*, *Peneaus monodon*, and *Metapeneaus ensis* were also included. This figure was reproduced and slightly modified from Kawato et al., 2019, J Virol 93:e01144-18, https://doi.org/10.1128/JVI.01144-18, with permission from the authors and the American Society for Microbiology.

**Fig. 2:**
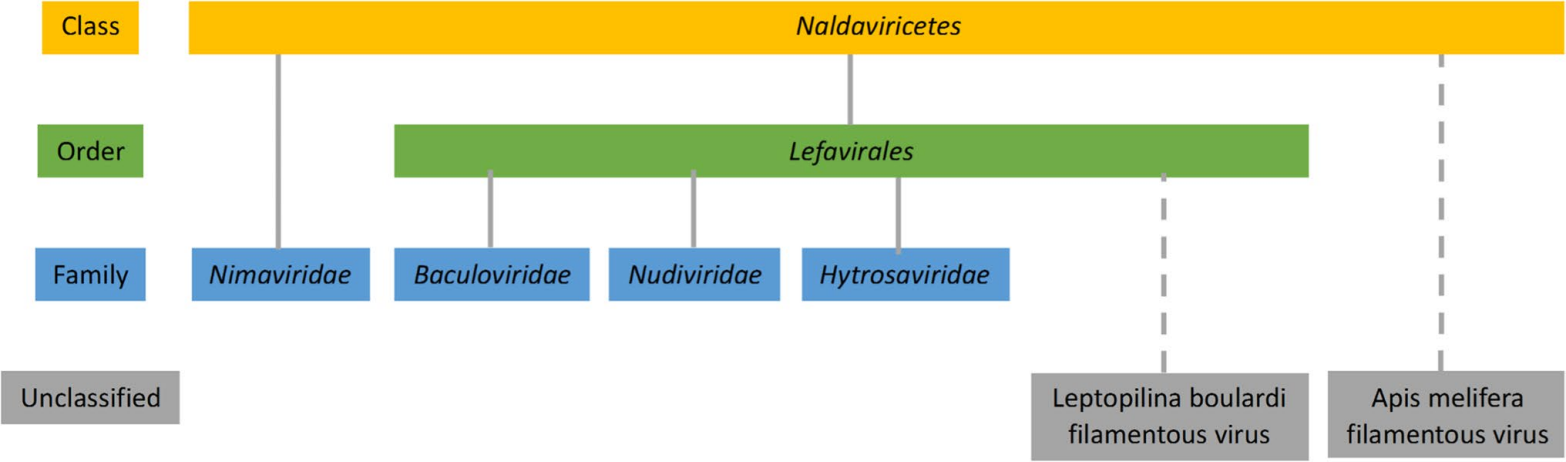
Taxonomic hierarchy of families of nuclear arthropod large DNA viruses. A new class, *Naldaviricetes*, was established for classification of the viruses in the four currently established families *Baculoviridae*, *Nudiviridae*, *Hytrosaviridae*, and *Nimaviridae. *A new order, *Lefavirales*, was introduced to include three of these families. The two viruses indicated at the bottom of the figure (Apis mellifera filamentous virus [3, 10] and Leptopilina boulardi filamentous virus [15]) are currently unclassified, but based on their genome content, they bear the hallmarks of members of the taxa *Naldaviricetes* and *Lefavirales*, respectively.

Errors in Table 3 were corrected. Revised version updated here.

**Table 3:** List of currently valid lefaviral binomial species names along with common virus names and their abbreviations. Please note that virus names will remain unaffected by recent nomenclatural changes

Family/genus	Binominal species name	Exemplar/common virus name	Abbreviation
**Family* Baculoviridae***			
*Alphabaculovirus*	*Alphabaculovirus adhonmai*	Adoxophyes honmai nucleopolyhedrovirus	AdhoNPV
	*Alphabaculovirus agipsilonis*	Agrotis ipsilon multiple nucleopolyhedrovirus	AgipNPV
	*Alphabaculovirus agsegetum*	Agrotis segetum nucleopolyhedrovirus A	AgseNPV-A
	*Alphabaculovirus alteragsegetum*	Agrotis segetum nucleopolyhedrovirus B	AgseNPV-B
	*Alphabaculovirus anpernyi*	Antheraea pernyi nucleopolyhedrovirus	AnpeNPV
	*Alphabaculovirus angemmatalis*	Anticarsia gemmatalis multiple nucleopolyhedrovirus	AgMNPV
	*Alphabaculovirus ardigrammae*	Artaxa digramma nucleopolyhedrovirus	ArdiNPV
	*Alphabaculovirus aucalifornicae*	Autographa californica multiple nucleopolyhedrovirus	AcMNPV
	*Alphabaculovirus bomori*	Bombyx mori nucleopolyhedrovirus	BmNPV
	*Alphabaculovirus busuppressariae*	Buzura suppressaria nucleopolyhedrovirus	BuzuNPV
	*Alphabaculovirus capomonae*	Catopsilia pomona nucleopolyhedrovirus	CapoNPV
	*Alphabaculovirus chofumiferanae*	Choristoneura fumiferana multiple nucleopolyhedrovirus	CfMNPV
	*Alphabaculovirus alterchofumiferanae*	Choristoneura fumiferana DEF multiple nucleopolyhedrovirus	CfMNPV-DEF
	*Alphabaculovirus chomurinanae*	Choristoneura murinana nucleopolyhedrovirus	ChmuNPV
	*Alphabaculovirus chorosaceanae*	Choristoneura rosaceana nucleopolyhedrovirus	ChroNPV
	*Alphabaculovirus chrychalcites*	Chrysodeixis chalcites nucleopolyhedrovirus	ChchNPV
	*Alphabaculovirus chrincludentis*	Chrysodeixis includens nucleopolyhedrovirus	ChinNPV
	*Alphabaculovirus clabilineatae*	Clanis bilineata nucleopolyhedrovirus	ClbiNPV
	*Alphabaculovirus covestigialis*	Condylorrhiza vestigialis nucleopolyhedrovirus	CovoNPV
	*Alphabaculovirus crypeltasticae*	Cryptophlebia peltastica nucleopolyhedrovirus	CrpeNPV
	*Alphabaculovirus cycundantis*	Cyclophragma undans nucleopolyhedrovirus	CyunNPV
	*Alphabaculovirus dijunonis*	Dione juno nucleopolyhedrovirus	DijuNPV
	*Alphabaculovirus ecobliquae*	Ectropis obliqua nucleopolyhedrovirus	EcobNPV
	*Alphabaculovirus eppostvittanae*	Epiphyas postvittana nucleopolyhedrovirus	EppoNPV
	*Alphabaculovirus eupseudoconspersae*	Euproctis pseudoconspersa nucleopolyhedrovirus	EupsNPV
	*Alphabaculovirus helarmigerae*	Helicoverpa armigera nucleopolyhedrovirus	HearNPV
	*Alphabaculovirus heleucae*	Hemileuca species nucleopolyhedrovirus	HespNPV
	*Alphabaculovirus hycuneae*	Hyphantria cunea nucleopolyhedrovirus	HycuNPV
	*Alphabaculovirus hytalacae*	Hyposidra talaca nucleopolyhedrovirus	HytaNPV
	*Alphabaculovirus lafiscellariae*	Lambdina fiscellaria nucleopolyhedrovirus	LafiNPV
	*Alphabaculovirus leseparatae*	Leucania separata nucleopolyhedrovirus	LeseNPV
	*Alphabaculovirus lonobliquae*	Lonomia obliqua nucleopolyhedrovirus	LoobNPV
	*Alphabaculovirus lydisparis*	Lymantria dispar multiple nucleopolyhedrovirus	LdMNPV
	*Alphabaculovirus lyxylinae*	Lymantria xylina nucleopolyhedrovirus	LyxyNPV
	*Alphabaculovirus mabrassicae*	Mamestra brassicae multiple nucleopolyhedrovirus	MbMNPV
	*Alphabaculovirus maconfiguratae*	Mamestra configurata nucleopolyhedrovirus A	MacoNPV-A
	*Alphabaculovirus altermaconfiguratae*	Mamestra configurata nucleopolyhedrovirus B	MacoNPV-B
	*Alphabaculovirus mavitratae*	Maruca vitrata nucleopolyhedrovirus	MaviNPV
	*Alphabaculovirus myunipunctae*	Mythimna unipuncta nucleopolyhedrovirus A	MyunNPV-A
	*Alphabaculovirus altermyunipunctae*	Mythimna unipuncta nucleopolyhedrovirus B	MyunNPV-B
	*Alphabaculovirus opbrumatae*	Operophtera brumata nucleopolyhedrovirus	OpbrNPV
	*Alphabaculovirus orleucostigmae*	Orgyia leucostigma nucleopolyhedrovirus	OrleNPV
	*Alphabaculovirus orpseudotsugatae*	Orgyia pseudotsugata multiple nucleopolyhedrovirus	OPMNPV
	*Alphabaculovirus oxochraceae*	Oxyplax ochracea nucleopolyhedrovirus	OxocNPV
	*Alphabaculovirus pesauciae*	Peridroma saucia nucleopolyhedrovirus	PesaNPV
	*Alphabaculovirus peluscae*	Perigonia lusca nucleopolyhedrovirus	PeluNPV
	*Alphabaculovirus ranus*	Rachiplusia nu nucleopolyhedrovirus	RanuNPV
	*Alphabaculovirus speridaniae*	Spodoptera eridania nucleopolyhedrovirus 251	SperNPV-251
	*Alphabaculovirus altersperidaniae*	Spodoptera eridania nucleopolyhedrovirus -CNPSo-165	SperNPV-CNPSo-165
	*Alphabaculovirus spexemptae*	Spodoptera exempta nucleopolyhedrovirus	SpexNPV
	*Alphabaculovirus spexiguae*	Spodoptera exigua multiple nucleopolyhedrovirus A	SeMNPV-A
	*Alphabaculovirus alterspexiguae*	Spodoptera exigua multiple nucleopolyhedrovirus B	SeMNPV-B
	*Alphabaculovirus spofrugiperdae*	Spodoptera frugiperda multiple nucleopolyhedrovirus	SfMNPV
	*Alphabaculovirus splittoralis*	Spodoptera littoralis nucleopolyhedrovirus	SpliNPV
	*Alphabaculovirus spliturae*	Spodoptera litura nucleopolyhedrovirus	SpltNPV
	*Alphabaculovirus sujujubae*	Sucra jujuba nucleopolyhedrovirus	SujuNPV
	*Alphabaculovirus thorichlaceae*	Thysanoplusia orichalcea nucleopolyhedrovirus	ThohNPV
	*Alphabaculovirus trini*	Trichoplusia ni single nucleopolyhedrovirus	TnSNPV
	*Alphabaculovirus urprotei*	Urbanus proteus nucleopolyhedrovirus	UrprNPV
	*Alphabaculovirus wisignatae*	Wiseana signata nucleopolyhedrovirus	WisiNPV
*Betabaculovirus*	*Betabaculovirus adoranae*	Adoxophyes orana granulovirus	AdorGV
	*Betabaculovirus agsegetum*	Agrotis segetum granulovirus	AgseGV
	*Betabaculovirus arrapae*	Artogeia rapae granulovirus	ArraGV
	*Betabaculovirus chofumiferanae*	Choristoneura fumiferana granulovirus	CfGV
	*Betabaculovirus clanachoretae*	Clostera anachoreta granulovirus	ClanGV
	*Betabaculovirus clanastomosis*	Clostera anastomosis granulovirus A	ClasGV-A
	*Betabaculovirus alterclanastomosis*	Clostera anastomosis granulovirus B	ClasGV-B
	*Betabaculovirus cnamedinalis*	Cnaphalocrocis medinalis granulovirus	CnmeGV
	*Betabaculovirus cryleucotretae*	Cryptophlebia leucotreta granulovirus	CrleGV
	*Betabaculovirus cypomonellae*	Cydia pomonella granulovirus	CpGV
	*Betabaculovirus disaccharalis*	Diatraea saccharalis granulovirus	DisaGV
	*Betabaculovirus epaporemae*	Epinotia aporema granulovirus	EpapGV
	*Betabaculovirus erellonis*	Erinnyis ello granulovirus	ErelGV
	*Betabaculovirus habrilliantis*	Harrisina brillians granulovirus	HabrGV
	*Betabaculovirus helarmigerae*	Helicoverpa armigera granulovirus	HearGV
	*Betabaculovirus hycuneae*	Hyphantria cunea granulovirus	HycuNPV
	*Betabaculovirus lacoleraceae*	Lacanobia oleracea granulovirus	LaolGV
	*Betabaculovirus molatipedis*	Mocis latipes granulovirus	MolaGV
	*Betabaculovirus myunipunctae*	Mythimna unipuncta granulovirus A	MyunGV-A
	*Betabaculovirus altermyunipunctae*	Mythimna unipuncta granulovirus B	MyunGV-B
	*Betabaculovirus maphaseoli*	Matsumuraeses phaseoli granulovirus	MaphGV
	*Betabaculovirus phoperculellae*	Phthorimaea operculella granulovirus	PhopGV
	*Betabaculovirus plinterpunctellae*	Plodia interpunctella granulovirus	PlinGV
	*Betabaculovirus pluxylostellae*	Plutella xylostella granulovirus	PlxyGV
	*Betabaculovirus spofrugiperdae*	Spodoptera frugiperda granulovirus	SfGV
	*Betabaculovirus spliturae*	Spodoptera litura granulovirus	SpltGV
	*Betabaculovirus trini*	Trichoplusia ni granulovirus	TnGV
	*Betabaculovirus xecnigri*	Xestia c-nigrum granulovirus	XecnGV
*Gammabaculovirus*	*Gammabaculovirus nelecontei*	Neodiprion lecontei nucleopolyhedrovirus	NeleNPV
	*Gammabaculovirus nesertiferis*	Neodiprion sertifer nucleopolyhedrovirus	NeseNPV
*Deltabaculovirus*	*Deltabaculovirus cunigripalpi*	Culex nigripalpus nucleopolyhedrovirus	CuniNPV
**Family* Nudiviridae***			
*Alphanudivirus*	*Alphanudivirus droinnubilae*	Drosophila innubila nudivirus	DiNV
	*Alphanudivirus dromelanogasteris*	Kallithea virus	KV
	*Alphanudivirus alterdromelanogasteris*	Tomelloso virus	TNV
	*Alphanudivirus tertidromelanogasteris*	Esparto virus	ENV
	*Alphanudivirus quartudromelanogasteris*	Mauternbach virus	MNV
	*Alphanudivirus grybimaculati*	Gryllus bimaculatus nudivirus	GbNV
	*Alphanudivirus oryrhinocerotis*	Oryctes rhinoceros nudivirus	OrNV
*Betanudivirus*	*Betanudivirus hezeae*	Heliothis zea nudivirus	HzNV
*Gammanudivirus*	*Gammanudivirus hogammari*	Homarus gammarus nudivirus	HgNV
	*Gammanudivirus pemonodonis*	Penaeus monodon nudivirus	PmNV
	*Gammanudivirus cracrangonis*	Crangon crangon nudivirus	CcNV
	*Gammanudivirus cameanadis*	Carcinas meneas nudivirus	CmPV
*Deltanudivirus*	*Deltanudivirus tipoleraceae*	Tipula oleracea nudivirus	ToGV
**Family* Hytrosaviridae***			
*Glossinavirus*	*Glossinavirus glopallidipedis*	Glossina pallipides salivery gland hypertrophy virus	GpSGHV
*Muscavirus*	*Muscavirus musdomesticae*	Musca domestica salivery gland hypertrophy virus	MdSGHV

The original article has been corrected.

