# Supplementary material for: Developments in the classification and nomenclature of arthropod-infecting large DNA viruses that contain pif genes
Source: Arch Virol. 2023 Jun 14;168(7):182. doi: 10.1007/s00705-023-05793-8 (PMC10271883; doi:10.1007/s00705-023-05793-8)
Supplement: Supplementary file 1 — Supplementary Material 1 [file 705_2023_5793_MOESM1_ESM.docx]

**Supplementary Table S1** Explanations of particular epithets for species in the order *Lefavirales*

| **Binomial species name** | **Exemplar/Common virus name** | **Explanation for epithet composition** |
| --- | --- | --- |
| *Alphabaculovirus aucalifornicae* | *Autographa californica multiple nucleopolyhedrovirus* | Two first letters of the host’s genus: Au from *Autographa*, connected to the genitive of the host’s epithet: *californicae* |
| *Alphabaculovirus altermaconfiguratae* | *Mamestra configurata nucleopolyhedrovirus B* | This is the second species recognised, so it has the prefix “alter”” before the rest of the epithet, derived from “Ma” after the host genus name *Mamestra*, followed by the host’s epithet in genitive form: *configuratae*. |
| *Alphabaculovirus adhonmai* | *Adoxophyes honmai nucleopolyhedrovirus* | “honmai” evidently came from Honma, a Japanese clan name, which was presumably changed to the nominative honmaus and from there to the genitive honmai for the species epithet of this tortricid moth. So honmai is already in the genitive form. |
| *Alphabaculovirus trini* | *Trichoplusia ni nucleopolyhedrovirus* | “ni” in the host’s name appears to be derived from the lowercase Greek letter “nu” (n), a pattern displayed on the wings of this moth. The ‘ni” might be a genitive form of the nominative form “nu” and was kept as it is. |
| *Alphabaculovirus bomori* | *Bombyx mori nucleopolyhedrovirus* | “mori” may have a non-Latin root in the Japanese word “mori” meaning forest. In Latin it already sounds like a genitive, so it has not been changed. |
| *Glossinavirus glopallidipedis* | *Glossina hytrosavirus* | The epithet of this virus species in the family *Hytrosaviridae* is derived from the host *Glossina pallidipes*. Pallidipes meaning something like “pale-footed”, and the genitive of pes is pedis. |
